# Post-translational negative feedback loops are sufficient to coordinate synthesis of the gram-negative envelope during steady-state growth

**DOI:** 10.1128/mbio.01199-26

**Published:** 2026-07-02

**Authors:** Jaïrus Beije, Gregory Bokinsky

**Affiliations:** 1 Department of Bionanoscience, Kavli Institute of Nanoscience, Delft University of Technology 2860, Delft, the Netherlands; The University of Texas Health Science Center at Houston John P. and Katherine G. McGovern Medical School, Houston, Texas, USA

**Keywords:** cell envelope, phospholipids, metabolic regulation, lipopolysaccharide, membrane biogenesis

## Abstract

How gram-negative bacteria coordinate the synthesis of their multilayered envelopes is a long-standing fundamental question. We compile protein and metabolite measurements obtained from *Escherichia coli* to eliminate mechanisms that do not coordinate envelope synthesis during steady-state growth. These measurements reveal that envelope synthesis pathway expression and envelope precursor concentrations are both stable across growth rates, thus eliminating enzyme levels and metabolite levels as coordination mechanisms. We propose instead that envelope assembly pathways are coordinated by post-translational mechanisms that control a small number of enzymes and transport proteins, which in turn control upstream synthesis pathways via classic negative feedback. We further hypothesize that many signals that have been proposed to directly regulate envelope synthesis pathways act indirectly via known negative feedback loops.

## OPINION/HYPOTHESIS

Gram-negative bacteria enclose themselves within a complex multilayered envelope of phospholipids, peptidoglycan, and lipopolysaccharides (LPSs). Each layer is assembled from precursors that are synthesized in the cytoplasm. As the envelope assembly and precursor synthesis pathways of model organisms are well described, current research focuses on understanding how the layers are built in a coordinated manner and in correct proportions (1). Researchers in the cell envelope field approach this problem by asking questions such as “How is LPS synthesis coordinated with phospholipid synthesis?” or “How is envelope biogenesis coordinated with cell growth?” Comprehensive reviews on this topic give the impression that envelope assembly requires a complicated scheme of many interactions between proteins, metabolites, transcription factors, and other signals. Alternatively, if envelope assembly is considered as a metabolic pathway, this problem becomes a specific case of the general question “How do living cells regulate metabolic pathways?” This question can be addressed by measuring intracellular concentrations of pathway enzymes and metabolites across a range of conditions. In this spirit, we examine published proteome and metabolome data sets obtained from *Escherichia coli* during steady-state growth (2–5) to evaluate mechanisms of envelope synthesis regulation.

## HOW DO CELLS CONTROL SYNTHESIS RATES DURING STEADY-STATE GROWTH?

**Fig 1:**
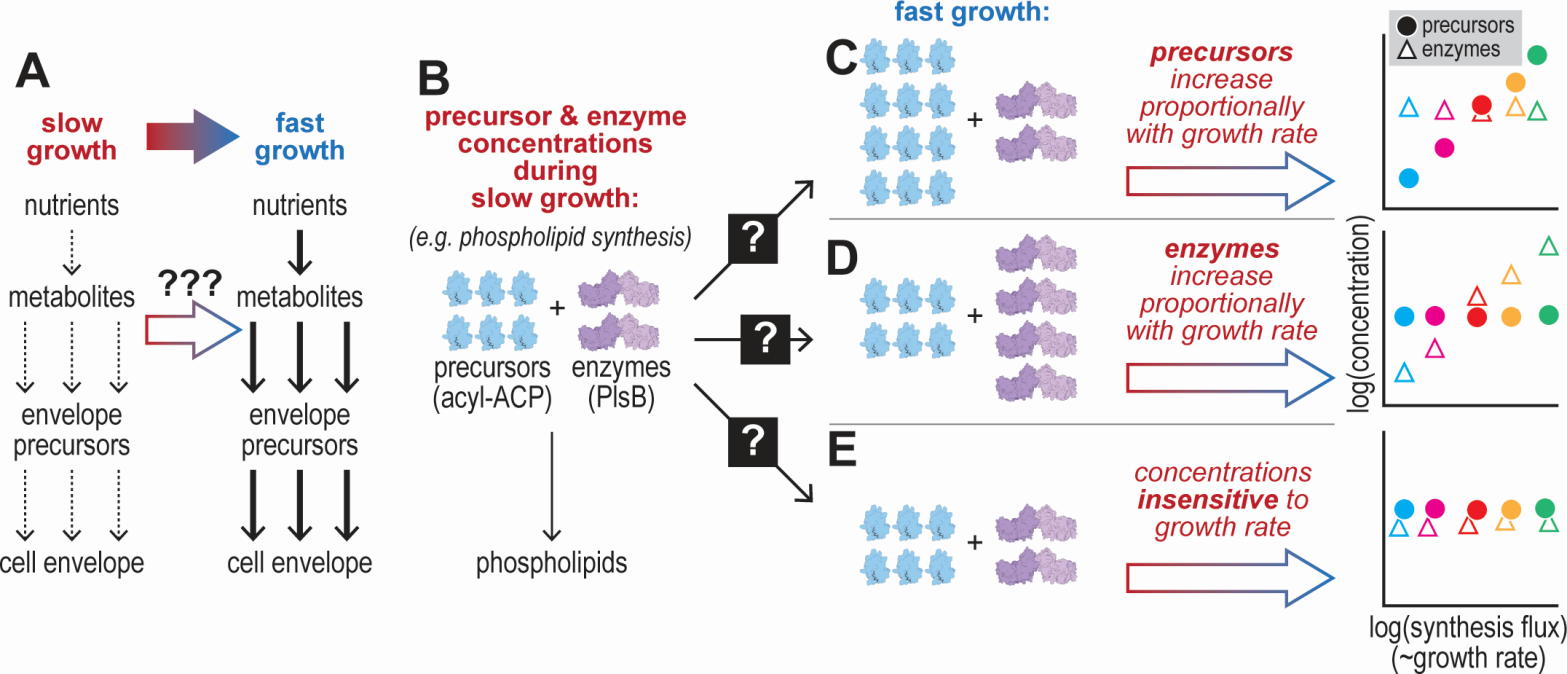
Inferring mechanisms of envelope biogenesis coordination by quantifying enzymes, metabolites, and precursors. (**A**) Envelope synthesis is synchronized with growth rate. (**B**) Synchronization mechanisms can be inferred by correlating metabolite and enzyme concentrations with growth rate over a range of conditions. For example, phospholipid synthesis regulation mechanisms can be inferred by correlating concentrations of phospholipid precursors and synthesis enzymes (e.g., PlsB) with phospholipid flux. These correlations indicate mechanisms such as control by substrate concentrations (**C**), enzyme concentrations (**D**), or concentration-independent mechanisms, including post-translational regulation (**E**). Panels C to E are inspired by reference 6.

An *E. coli* NCM3722 cell in amino acid-supplemented glucose medium doubles every 30 minutes and therefore needs to synthesize phospholipids, peptidoglycan, and LPS at faster rates than when doubling every ~90 minutes in acetate minimal medium. How does *E. coli* adjust envelope synthesis rates to match its growth rate (Fig. 1A)?

Product synthesis rates may be adjusted by changes in concentrations of reactants, enzymes, or regulators, or by changing the activities of pathway enzymes via competitive inhibition or allosteric interactions. The mechanisms used can be inferred by comparing the concentrations of pathway reactants, enzymes, and regulators across a range of steady-state growth conditions (6, 7) (Fig. 1B–E). When the concentration of a pathway substrate (Fig. 1C) or enzyme (Fig. 1D) varies monotonically and proportionally with pathway flux, it suggests (but does not prove) that the rate of the reaction is controlled by concentration changes. For instance, the protein translation rate is tuned primarily by varying ribosome abundance via transcription (8), whereas the rates of fatty acid elongation reactions rise with the concentration of the substrate malonyl-ACP (2). While correlations cannot unambiguously establish how flux is controlled, the absence of correlation can definitively rule out specific mechanisms: for instance, if enzyme or substrate concentrations remain stable across all growth rates (Fig. 1E) or vary in an incoherent manner, then the synthesis rate during growth is not controlled by concentration, indicating a different mechanism such as allosteric inhibition. So what does not control envelope synthesis rates during steady-state growth?

### Enzyme concentrations are not used to control envelope synthesis rates

**Fig 2:**
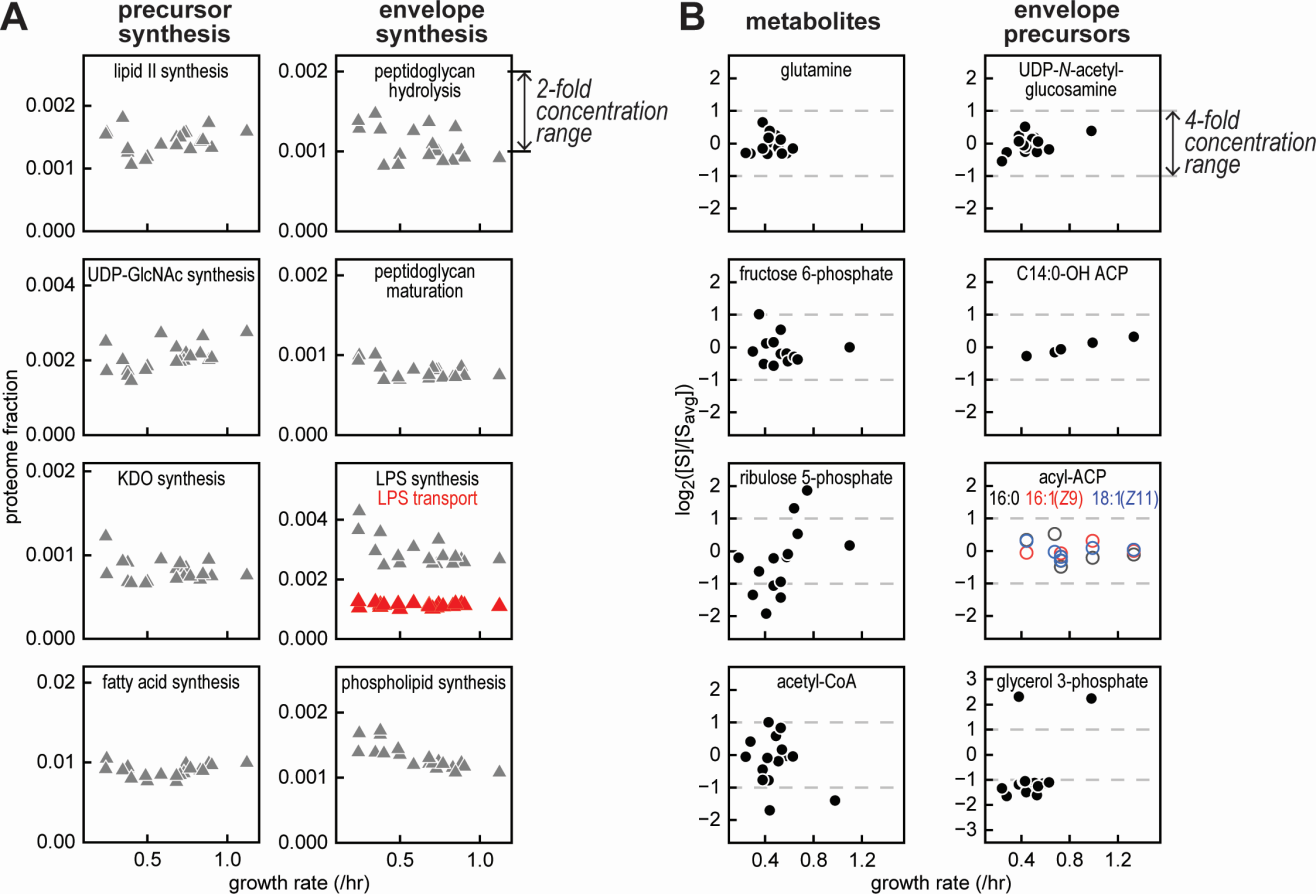
Concentrations of pathway enzymes, metabolites, and envelope precursors do not control envelope biosynthesis. (**A**) Proteome fractions of precursor synthesis and envelope assembly pathways. Pathway fractions are obtained by summing proteome fractions of individual enzymes within each pathway (enzymes grouped by pathway as depicted in Fig. S1 to S3). (**B**) Concentrations of metabolites and envelope precursors. Values are divided by the average concentration and log_2_ normalized. Proteome data are from reference 4; UDP-GlcNAc, glutamine, acetyl-CoA, and G3P measurements from reference 3; fructose-6-phosphate and ribulose-5-phosphate from reference 5; C14:0-OH ACP and long-chain acyl-ACP from reference 2.

For *in vitro* reactions, increasing the enzyme concentration usually increases the product formation rate. By this logic, *E. coli* might coordinate synthesis rates by tuning enzyme concentrations via transcriptional or translational regulation (or both). Just as the concentrations of ribosomes and translation cofactors vary in parallel with growth rate, *E. coli* might likewise vary expression of envelope synthesis pathways to adjust envelope assembly rates. However, unlike ribosomes and translation cofactors, the proteome fractions occupied by envelope synthesis pathways do not increase with growth (Fig. 2A) nor do concentrations of individual pathway enzymes (Fig. S1 to S3) (2, 4). Even the concentrations of enzymes that are regulated by proteolysis do not increase with growth (discussed further in Text S1). Thus, available data indicate that envelope synthesis rates are not coordinated by enzyme concentrations. Instead, *E. coli* regulates protein levels to maintain a fixed envelope synthesis capacity regardless of growth rate, an inefficient strategy during slow growth but advantageous in changing environments. Aside from envelope stress responses such as LPS modification pathways (9), envelope biogenesis in *E. coli* is coordinated primarily—perhaps exclusively—via post-translational mechanisms.

### Metabolite concentrations and envelope precursor concentrations do not determine envelope synthesis rates

An alternative mechanism for controlling envelope synthesis rates is to vary concentrations of envelope precursors such as fatty acid thioesters, glycerol-3-phosphate, and UDP-*N*-acetylglucosamine. This possibility is intuitively appealing because *in vitro* rates of enzymatic reactions are sensitive to substrate concentrations. However, *in vivo* measurements indicate that envelope precursor concentrations are either stable or increase only slightly in parallel with growth rate (Fig. 2B). Even massive increases in envelope precursor concentrations (e.g., glycerol-3-phosphate during glycerol catabolism) do not accelerate envelope synthesis (2). Therefore, the synthesis rates of cell envelope components are not coordinated by precursor concentrations. Strikingly, precursor levels are generally far more stable than upstream metabolites. For example, acetyl-CoA varies more than fourfold and shows no correlation with growth rate. These variations indicate that upstream metabolites also do not directly coordinate envelope synthesis.

### Envelope assembly reactions control envelope precursor synthesis

How might *E. coli* coordinate envelope biogenesis without varying precursors, metabolites, or enzyme levels? As described below, we hypothesize that cell envelope assembly pathways are regulated by post-translational negative feedback. This mechanism has been experimentally established for most of the pathways that supply precursors that are consumed by envelope assembly (e.g., fatty acyl-ACP, glycerol-3-phosphate, and UDP-*N*-acetylglucosamine). These precursors inhibit their own synthesis, often via allosteric interactions (mechanisms are compiled in Text S2). Negative feedback stabilizes precursor levels across growth rates, preventing both precursor excess and precursor depletion. This insulates the precursor supply from disturbances, including variations in upstream metabolite concentrations, as illustrated by comparing the stability of acyl-ACP against the variability of acetyl-CoA (Fig. 2B). Furthermore, negative feedback ensures that the enzymes that consume the precursors—and not the pathways that produce the precursors—exert control over precursor synthesis. The “rate-limiting step” of precursor synthesis is the reaction that consumes the precursor (10).

To illustrate this counterintuitive principle with an example, the first enzyme of the fatty acid synthesis pathway (acetyl-CoA carboxylase [ACC]) is inhibited by the product of the fatty acid pathway, long-chain acyl-ACP (2, 11, 12). Because this inhibitor is primarily consumed by the phospholipid synthesis enzyme PlsB, it is PlsB that determines the concentration of the ACC inhibitor and thereby controls ACC activity. Negative feedback couples acyl-ACP synthesis initiation to phospholipid synthesis: when phospholipid synthesis slows, acyl-ACP accumulates, and acyl-ACP synthesis initiation is inhibited. Conversely, when phospholipid synthesis accelerates, acyl-ACP is depleted, thus relieving ACC inhibition and increasing acyl-ACP synthesis. Negative feedback thus synchronizes precursor synthesis with envelope assembly.

### Many responses that suggest the existence of direct regulatory interactions are actually caused by negative feedback

Negative feedback is simple, ubiquitous, and widely acknowledged as a regulatory motif at transcriptional, translational, and post-translational levels. However, the versatility of negative feedback is poorly appreciated. Negative feedback is rarely invoked to explain unusual responses or coordination of synthesis pathways. Instead, unexpected experimental observations often inspire proposals for additional interactions between enzymes or other signals. We predict that many of these proposed interactions are unnecessary. This is because despite its simplicity, negative feedback generates counterintuitive responses. Many triggers proposed to directly inhibit a synthesis pathway often instead inhibit consumption of the pathway product. Since inhibiting product consumption causes product accumulation, product synthesis is inhibited due to negative feedback. Thus, such triggers actually inhibit the pathway indirectly via negative feedback, not through a direct interaction with pathway enzymes.

Two cases illustrate how negative feedback masquerades as a direct interaction. As described above, fatty acid synthesis initiation by ACC is feedback-inhibited by the pathway product, acyl-ACP. The observation that high concentrations of the alarmone guanosine tetraphosphate (ppGpp) inhibit fatty acid synthesis led to the proposal that ppGpp directly inhibits ACC (13). However, ppGpp also suppresses phospholipid synthesis, leading to acyl-ACP accumulation. Eliminating long-chain acyl-ACP with a thioesterase relieved inhibition despite abundant ppGpp, demonstrating that ppGpp only indirectly inhibits ACC by increasing acyl-ACP levels (11). Likewise, the fatty acid catabolism intermediate acyl-CoA was proposed to slow fatty acid synthesis by inhibiting the enoyl-ACP reductase FabI (14). Our *in vivo* measurements of acyl-ACP pools demonstrated that acyl-CoA does not inhibit FabI but instead triggers acyl-ACP accumulation by competing for phospholipid synthesis enzymes PlsB and PlsC (15). Thus, neither ppGpp nor acyl-CoA inhibits fatty acid synthesis via a direct interaction with pathway enzymes but instead by increasing acyl-ACP and thus, via negative feedback.

### Two negative feedback loops are sufficient to coordinate LPS synthesis with growth

**Fig 3:**
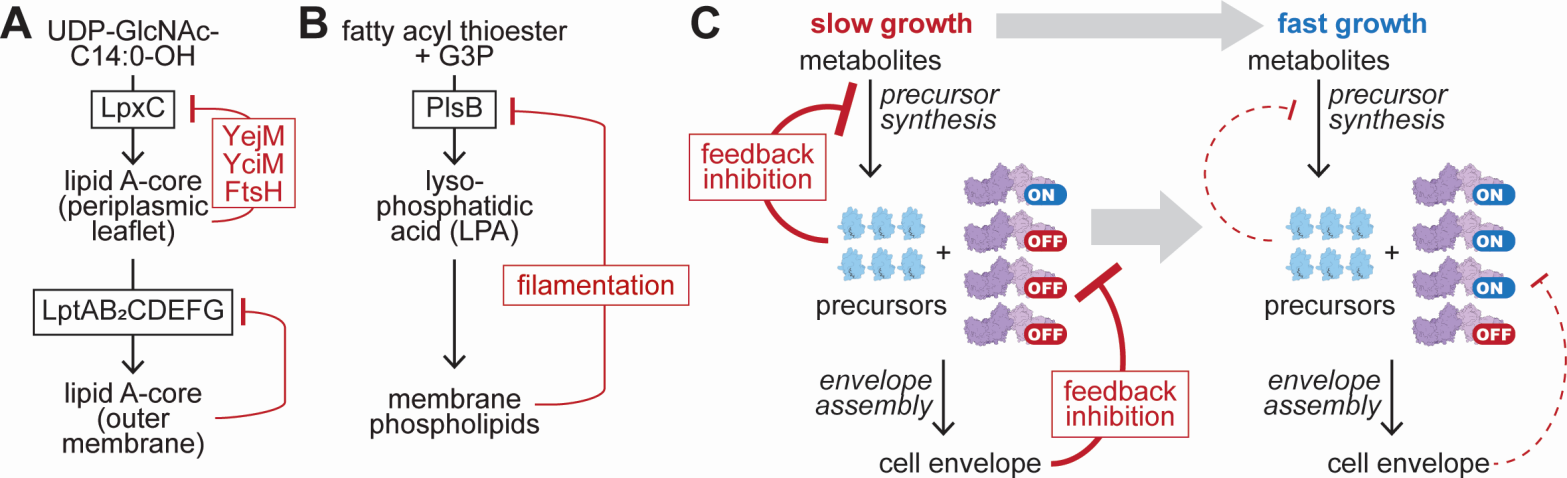
Serial negative feedback loops coordinate precursor synthesis and envelope assembly with growth. (**A**) Negative feedback loops acting on the LPS synthesis and transport pathways. Inhibition of the Lpt complex by outer membrane LPS coordinates LPS transport with outer membrane expansion, while inhibition of LpxC activity by the YejM-YciM-FtsH system maintains periplasmic LPS at non-toxic concentrations. (**B**) We predict that phospholipid accumulation along the inner membrane triggers PlsB to assemble into enzymatically inactive filaments. This interaction establishes a negative feedback loop regulating PlsB activity and thus coordinating phospholipid synthesis with growth. (**C**) An overall scheme of cell envelope synthesis regulation. Envelope precursor and pathway enzyme concentrations are maintained at fixed levels regardless of growth rate. Envelope synthesis is coordinated with growth rate by tuning the degree of negative feedback within each pathway.

Similarly, we hypothesize that many regulatory interactions proposed to control LPS synthesis will prove to act via negative feedback (16). LPS is synthesized in the cytoplasm and exported to the periplasm, where it accumulates to low levels before export to the outer membrane (Text S2). The abundance of periplasmic LPS is monitored in *E. coli* by the YejM-YciM-FtsH system (also known as the LapC-LapB-FtsH system), which controls the concentration of LpxC, the second enzyme of LPS synthesis, by proteolysis. Excess periplasmic LPS increases LpxC proteolysis and thereby slows LPS synthesis, while depletion of periplasmic LPS decreases LpxC proteolysis. By linking LpxC activity with periplasmic LPS abundance, the YejM-YciM-FtsH system establishes a classic negative feedback loop (Fig. 3A). Evaluating whether negative feedback alone is sufficient to stabilize periplasmic LPS levels may require experimental methods for quantifying periplasmic LPS as a high-turnover transport intermediate.

While the negative feedback loop established by YejM-YciM-FtsH is well accepted, its role during steady-state growth remains unclear, as surprisingly, LpxC concentrations do not increase with growth rate in any proteomics data we are aware of (Text S1). How then might YejM-YciM-FtsH regulate LpxC activity? One recent proposal has been inspired by the discovery that formation of the YciM-LpxC complex is sufficient to inhibit LpxC (17). As inactivation of LpxC by YciM is driven by periplasmic LPS accumulation, reversibly tuning LpxC inhibition by YciM (instead of adjusting LpxC concentration via proteolysis) may allow small variations in LPS initiation rates during steady-state growth. Tools for monitoring YciM-LpxC complex formation may help to untangle the roles of reversible LpxC inhibition and proteolysis by FtsH.

Since the outer membrane is ultimately where LPS ends up, how does periplasmic LPS respond to the demand for outer membrane LPS? *In vitro* experiments with reconstituted Lpt complexes suggest that LPS transport is feedback-inhibited by the abundance of LPS on the outer membrane (18). If this inhibition is present *in vivo*, it would establish a second negative feedback loop that communicates LPS demand from the outer membrane to the periplasmic LPS pool (Fig. 3A). We speculate that outer membrane expansion allows insertion of LPS, thereby relieving Lpt inhibition and favoring LPS transport. In such a scheme, the YejM-YciM-FtsH system does not coordinate LPS synthesis with growth but instead maintains periplasmic LPS concentration within a range that avoids disrupting the inner membrane while supporting outer membrane assembly.

## PHOSPHOLIPID SYNTHESIS IS REGULATED BY NEGATIVE FEEDBACK

Phospholipid synthesis is initiated by PlsB, which transfers an acyl chain from acyl-ACP or acyl-CoA to glycerol-3-phosphate to produce lysophosphatidic acid (Text S2). As the first enzyme of phospholipid synthesis, PlsB is the most likely target for allosteric regulation (2), but what coordinates PlsB activity and membrane synthesis with growth?

Overexpressed PlsB assembles into filaments that wrap helically around a phospholipid core (19). While PlsB filaments are enzymatically inactive, disassembling the filaments restores activity (20). How might PlsB filamentation establish a negative feedback loop and regulate phospholipid synthesis? Many metabolic enzymes reversibly assemble into inactive filaments when their products accumulate (21), thus establishing negative feedback. By analogy, PlsB filamentation may be triggered by surplus phospholipids. Filamentation would couple phospholipid synthesis with membrane expansion: growth stretches the membrane, dissolving inactive PlsB filaments into active PlsB monomers and thereby increasing phospholipid synthesis (Fig. 3B).

Our live-cell fluorescence imaging experiments support this filamentation hypothesis (22). GFP-tagged PlsB localizes as discrete foci when expressed at wild-type levels, consistent with filament formation. Inhibiting phospholipid synthesis disperses fluorescence, consistent with filament disassembly and release of PlsB inhibition. When phospholipid synthesis is restored, the PlsB foci reform after a period of growth. Thus, PlsB foci behave in a manner consistent with filaments assembling and disassembling in response to phospholipid abundance. Further experiments with filamentation-deficient PlsB mutants are required to determine whether filamentation establishes a negative feedback loop that ensures phospholipid homeostasis. We note that a negative feedback loop responsive to membrane electrostatic charge maintains phospholipid headgroup composition (Text S2).

### The peptidoglycan expansion rate is not determined by enzyme expression or peptidoglycan precursor supply

The “break-before-make” model of peptidoglycan expansion predicts that the steps controlled by growth are the hydrolysis reactions that allow monomer insertion by creating gaps in peptidoglycan strands (23, 24). Consequently, concentrations of the peptidoglycan precursor lipid II should not affect the rate of peptidoglycan mesh expansion. To our knowledge, there are no measurements of lipid II concentrations across growth rates. However, depletion of cell wall precursors does not slow surface growth in the short term, and overexpression of a peptidoglycan hydrolase transiently increases cell expansion (25). These findings support the break-before-make model and indicate that peptidoglycan expansion is not determined by regulation of enzyme expression or lipid II supply. Maintaining peptidoglycan precursors and pathway enzymes at stable levels across growth rates mirrors the strategy used by phospholipid and LPS synthesis pathways.

Since hydrolysis weakens the peptidoglycan mesh and thus threatens its structural integrity, peptidoglycan hydrolases must be tightly controlled. In a striking parallel with LPS synthesis, three endopeptidases (MepS, MepM, and MepH) are degraded by a periplasmic protease, Prc. Proteolysis is in turn mediated by an outer membrane lipoprotein, NlpI. While MepS proteolysis may determine the upper limit of MepS activity, a reversible MepS-NlpI association may also control MepS activity, just as YciM may reversibly inhibit LpxC. How lipoprotein association regulates peptidoglycan hydrolase activity will likely reveal how growth controls peptidoglycan expansion (24).

### A general model: envelope stretching allosterically controls the rate of envelope synthesis by relieving negative feedback

We propose that cell volume expansion allosterically activates specific enzymes and transporters: specifically, PlsB, the Lpt transport complex, and peptidoglycan hydrolases. These reactions in turn control the synthesis of envelope precursors by consuming the precursors, which relieves feedback inhibition of the upstream pathways. As both envelope precursor supply and enzyme levels are stable across steady-state growth rates, we predict that these allosteric control points will prove sufficient to coordinate envelope assembly during steady-state growth (Fig. 3C).

It is important to note that the mechanism of flux control is always condition dependent. The regulatory principles described here may not apply when growth is inhibited, when the cell envelope is stressed or damaged, for mutant strains, or during acute stress. For instance, many envelope precursors are depleted during stationary phase (3). When precursors are depleted, the post-translational signals that control envelope assembly during growth may not be active. Furthermore, not every gram-negative species may follow these principles (1, 16). Nevertheless, we hope to have demonstrated how existing tools clarify the search for regulatory mechanisms, and we hope to inspire researchers to consider negative feedback before proposing a novel regulatory interaction. We also hope to provoke experiments that test whether many proposed signals act via negative feedback. After all, eliminating potential regulatory schemes is just as important for understanding envelope biogenesis as is the discovery of novel interactions. Biology often appears complex because we overlook simplicity.

## Data Availability

All data are obtained from planktonic cultures of *E. coli* K12 derivatives. Data in Fig. 2 and supplemental figures are provided as an Excel file. Proteome data (excluding data from non-growing cultures) are taken from reference 4, which describes *E. coli* NCM3722 and derivatives in various media. Metabolite data are from references 3 and 5, which primarily used *E. coli* BW25113. Acyl-ACP data from reference 2 were obtained from *E. coli* NCM3722.

## SUPPLEMENTAL MATERIAL

Supplemental Material mbio.01199-26-s0001.pdf Fig. S1-S3, Texts S1 and S2, and supplemental references.

Source Data mbio.01199-26-s0002.xlsx Source data for Fig. 2 and Fig. S1-S3.

ASM does not own the copyrights to Supplemental Material that may be linked to, or accessed through, an article. The authors have granted ASM a non-exclusive, world-wide license to publish the Supplemental Material files. Please contact the corresponding author directly for reuse.
