# Supplementary material for: Post-translational negative feedback loops are sufficient to coordinate synthesis of the gram-negative envelope during steady-state growth
Source: mBio. 2026 Jul 2;17(8):e01199-26. doi: 10.1128/mbio.01199-26 (PMC13464355; doi:10.1128/mbio.01199-26)
Supplement: Supplementary file 1 [file mbio.01199-26-s0001.pdf]

Supplementary Information

Post-translational negative feedback loops are sufficient to coordinate synthesis of the Gram-negative envelope during steady-state growth

Jaïrus Beije, Gregory Bokinsky

Department of Bionanoscience, Kavli Institute of Nanoscience, Delft University of Technology, Delft, The Netherlands.

Materials and correspondence: g.e.bokinsky@tudelft.nl

Contents

Supplementary Information 1
Supplemental Figure 1 2
Supplemental Figure 2 3
Supplemental Figure 3 5
Supplemental Text 1 6
- The role of proteolysis in controlling enzyme concentrations during steady-state growth is unclear 6
- On the importance of rapid quenching for quantifying proteins regulated by proteolysis 7

Supplemental Text 2 8
- Fatty acid synthesis 8
- Glycerol-3-phosphate synthesis 9
- Phospholipid headgroup composition is maintained by negative feedback 10
- UDP-*N*-acetyl glucosamine synthesis 11
- LPS synthesis 12
- CMP-3-deoxy-D-*manno*-octulosonate (KDO) synthesis 13
- LPS core synthesis 13
- Peptidoglycan synthesis 14
- MurA is inhibited by UDP-MurNAc 14
- Glutamate racemase MurI is activated by UDP-MurNAc-L-Ala 14
- Lipid II synthesis is feedback-inhibited across the inner membrane by periplasmic lipid II in *Pseudomonas aeruginosa* 14

Supplemental References 16

Supplemental Figure 1

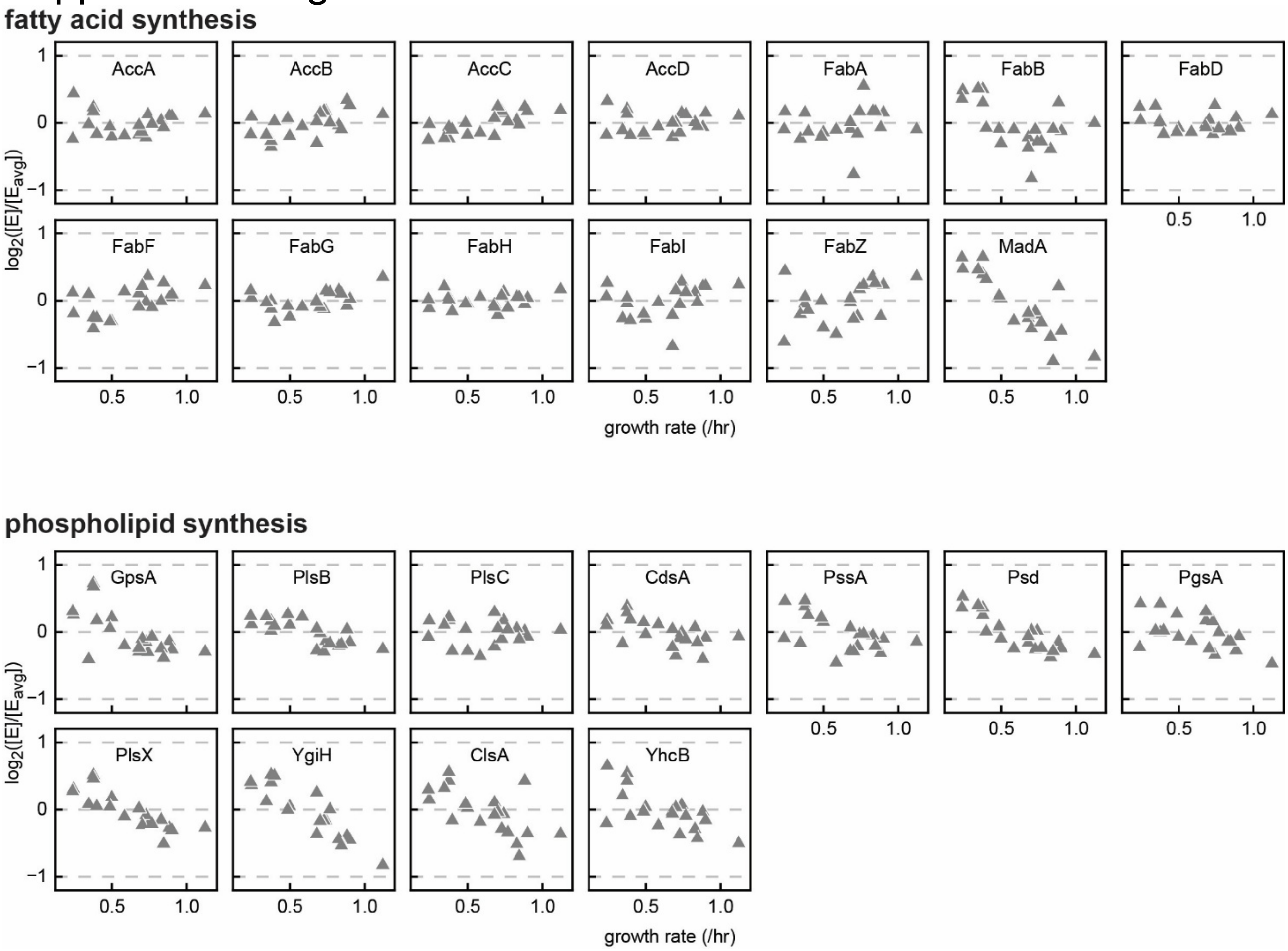

Supplemental Figure 1. Proteome mass fractions of enzymes of fatty acid and phospholipid synthesis pathways during steady-state growth. Mass fraction of each enzyme is normalized to its average value over the selected conditions and log_2-transformed to depict trends.

Supplemental Figure 2

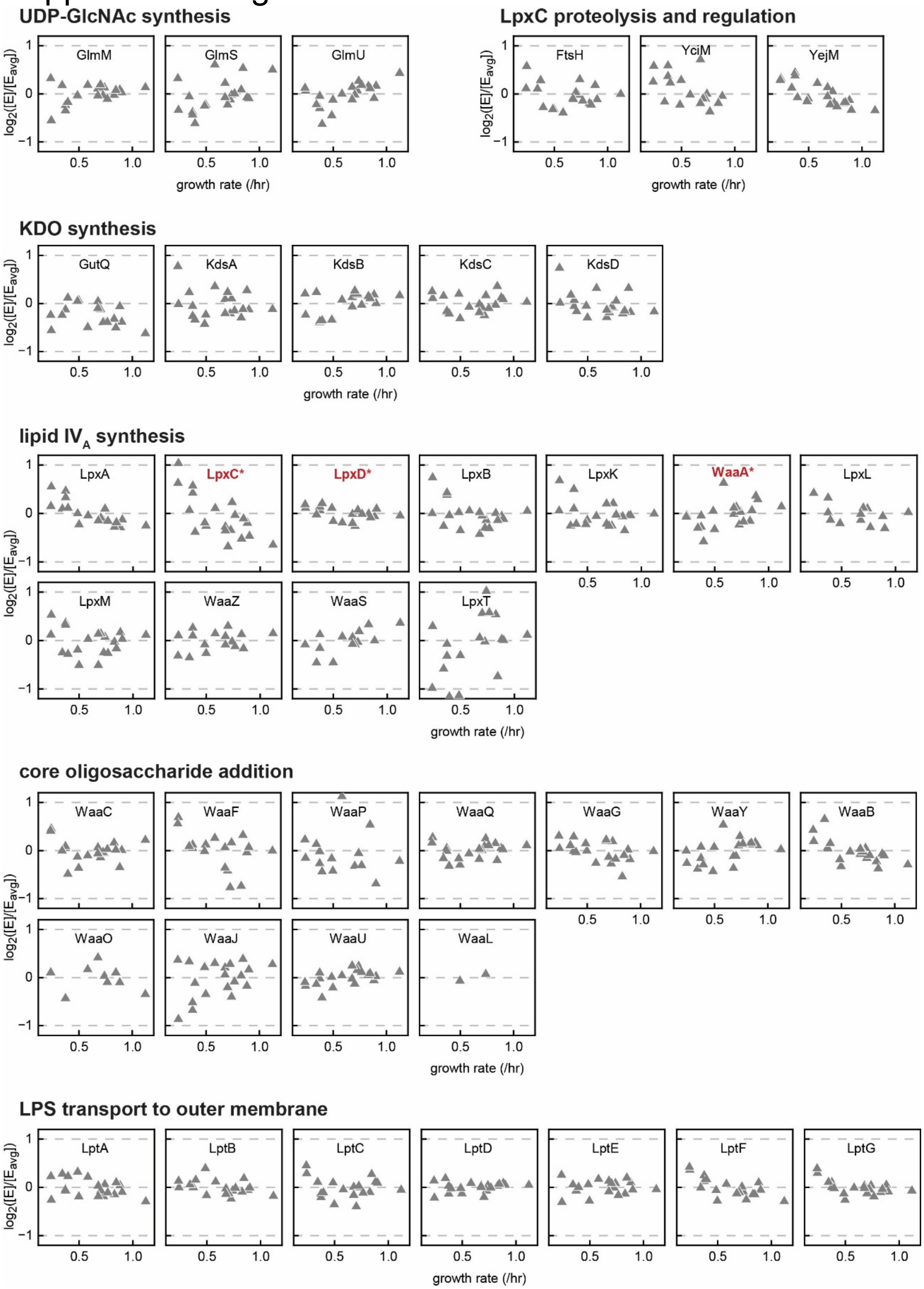

Supplemental Figure 2. Proteome mass fractions of enzymes of LPS precursor synthesis, LPS synthesis, and LPS transport pathways normalized as described in Supplemental Figure 1. Proteins known to be degraded by proteolysis (LpxC, LpxD, WaaA) are indicated with a red label and an asterisk.

Supplemental Figure 3

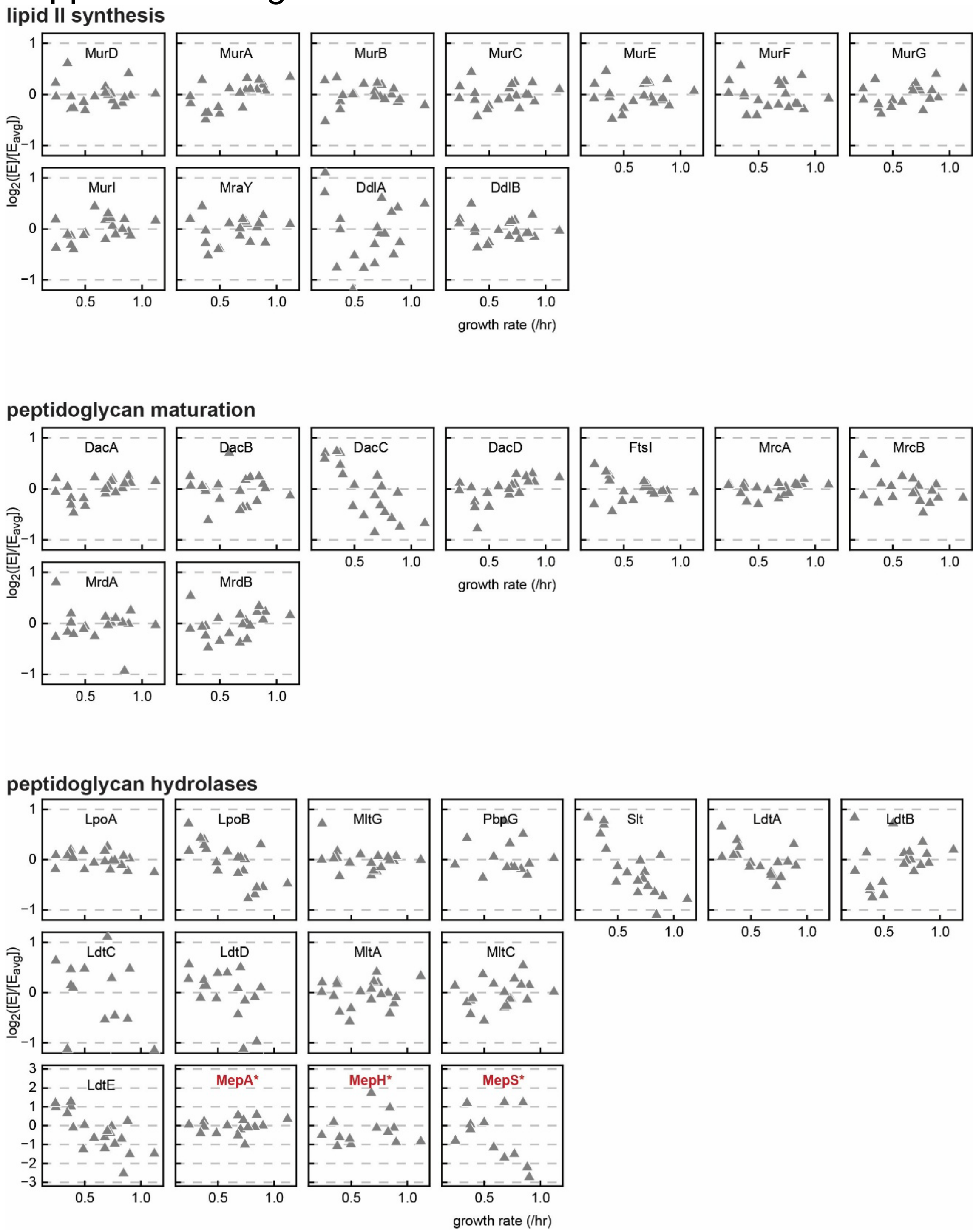

Supplemental Figure 3. Proteome mass fractions of enzymes of lipid II synthesis, lipid II transport, peptidoglycan maturase and peptidoglycan hydrolase pathwyas normalized as described in Supplemental Figure 1. Proteins known to be degraded by proteolysis (MepA, MepH, MepS) are indicated with a red label and an asterisk.

Supplemental Text 1

The role of proteolysis in controlling enzyme concentrations during steady-state growth is unclear

The discovery that inhibition of LPS synthesis increases LpxC expression via a transcription-independent mechanism revealed that LpxC is continuously proteolyzed and that the rate of proteolysis responds to LPS flux (1). The publication describing this discovery also predicted the existence of a negative feedback loop in which LPS demand influences LpxC activity. The workings of this negative feedback loop have been unraveled and are reviewed extensively elsewhere (2). Strong perturbations to LPS synthesis such as LpxC inhibition, FabZ overexpression (3) or LPS transport inhibition (4) are known to change LpxC concentrations in a homeostatic manner. A logical conclusion from such observations is that LpxC proteolysis would also cause LpxC concentrations to increase in parallel with growth rate. However, to our knowledge, a direct correlation between LpxC concentrations and growth rate has not been observed. As previously proposed (5) and as described in the main text, we hypothesize that reversible inhibition of LpxC by formation of the YciM-LpxC complex may be sufficient to maintain periplasmic LPS levels within a homeostatic range during steady-state growth, while dramatic changes in LPS demand (e.g. triggered by stress) are required to affect LpxC concentrations. Another possibility is that periplasmic LPS levels *do* vary somewhat across steady-state growth rates (i.e. they are *not* maintained at a fixed level) but that these small variations are not toxic or are at least insufficient to vary LpxC concentrations. As stated in the main text, direct measurements of periplasmic LPS are needed to illuminate how the YejM-YciM-FtsH feedback is used during steady-state growth.

What is the role of proteolysis not only for LpxC, but for other enzymes targeted by proteolysis (e.g. WaaA, LpxD, MepS, and AccA)? It may be that proteolysis is not used to vary steady-state flux (as is frequently assumed) but instead to ensure that a pathway is entirely inactive when it is no longer needed. Concentrations of proteins subject to constitutive proteolysis may also increase or decrease more rapidly than proteins dependent upon transcription. Further experiments comparing steady-state concentrations of these important proteins across growth conditions (and during dynamic conditions) are essential for determining the role of proteolysis.

On the importance of rapid quenching for quantifying proteins regulated by proteolysis

As metabolic reactions are extremely rapid (~seconds of faster), the metabolite pools in growing cells undergo constant turnover. Any perturbation in metabolic rates is likely to affect metabolite concentrations. These changes can be easily triggered by common laboratory steps such as pipetting from a well-mixed culture into a small tube, placing on ice, or (especially) centrifugation. These pitfalls are well-known in the metabolomics field and the importance of rapid quenching during sampling is widely emphasized (6). However, rapid quenching is less common in protocols for protein quantification. This is understandable as the concentrations of *most* proteins cannot change as rapidly as metabolites. However, for proteins regulated by constitutive proteolysis, rapid quenching may be important. Multiple proteins central to *E. coli* cell envelope biogenesis are known targets of proteolysis (most famously LpxC, but also MepS, AccA, and others). Unfortunately, the data set used here (Mori *et al.*) is generated using a protocol that does not rapidly quench cells, so it is possible that proteins subject to continuous degradation responded to sampling. For instance, LpxC concentrations appear to vary inversely with growth rate (**Supplemental Figure 2**). This result contradicts with our targeted proteomics data series obtained from rapidly-quenched samples, which found that LpxC concentrations do not vary with growth rate (7). Quantifying unstable proteins in samples that are rapidly quenched samples are needed to determine how such proteins vary with growth rate.

Supplemental Text 2

Inhibition of a synthesis pathway by its product has been demonstrated for many envelope precursor synthesis pathways. Here we review published work on the post-translational mechanisms regulating these pathways. While negative feedback is also a recurring motif in transcriptional and translational regulatory circuits and contributes to maintaining stable metabolite levels across growth rates, here we consider only mechanisms where metabolites act directly on enzymes (either competitive inhibition or allosteric inhibition).

Fatty acid synthesis

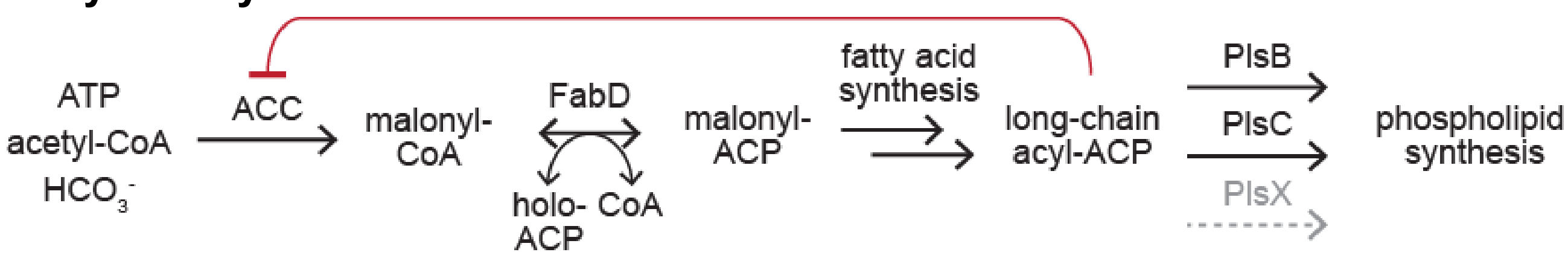

The first step of fatty acid synthesis is catalyzed by the acetyl-CoA carboxylase complex (ACC), which converts acetyl-CoA to malonyl-CoA in a biotin-dependent series of reactions that consume ATP and bicarbonate (8, 9). Next, the malonyl group is transferred from CoA to the phosphopantethiene thiol of acyl carrier protein (holo-ACP) by malonyl thiotransferase (FabD) (10, 11). Malonyl-ACP provides the carbon required for both fatty acid initiation and elongation reactions. Early studies found that fatty acid synthesis is tightly coupled to phospholipid synthesis. A mutant deficient in G3P (required for long-chain acyl-ACP consumption by the phospholipid synthesis pathway) accumulates abnormally long acyl-ACP and fatty acid synthesis is inhibited by ~90% when G3P is withheld (12). Cytoplasmic expression of the thioesterase TesA, which depletes acyl-ACP by hydrolyzing the thioester bond, relieves this inhibition. Furthermore, balanced expression of all four ACC subunits increased concentrations of malonyl-CoA by more than 100-fold but did not increase abundance of total phospholipids (13). Co-expression of TesA with ACC resulted in a 6-fold increase in free fatty acids and only a mild decrease in total phospholipid abundance. These results are consistent with acyl-ACP acting as a negative feedback inhibitor of ACC activity (14). TesA expression short-circuits the acyl-ACP negative feedback loop and is widely exploited in metabolic engineering to generate free fatty acids (15, 16).

Later biochemical experiments found that acyl-ACP species with a variety of chain lengths are equally capable of inhibiting ACC. In cell extracts, 40 μM acyl-ACP of all chain lengths tested (C4, C8, C12, C16, C16:1, C18:1, and C20:1) reduces ACC activity by roughly

65%, while holo-ACP did not inhibit ACC (17). However, further studies suggest that ACC regulation may not be simple. Initial characterization found both competitive and non-competitive inhibition by acyl-ACP (17). ACC inhibition exhibits hysteresis (the dependence of a state of a system on its history): varying the length of incubation of the purified ACC complex prior to the addition of palmitoyl-ACP affects the degree of inhibition observed (18). More recent work suggests that palmitoyl-ACP disrupts binding between the carboxyltransferase and the biotin carboxylase complexes (18).

A recent report described how the ACC complex assembles into filamentous structures, a phenomenon long known to exist for eukaryotic ACC (19, 20). In the presence of substrates, ACC assembles into well-ordered hollow tubes (21). While the substrate requirement for ordered assembly into filaments hints at a possible importance for activity, the physiological relevance of these filaments is yet to be determined. Nevertheless, the fact that *E. coli* ACC forms filaments is likely an essential feature to account for in models of ACC regulation. Structural studies of *E. coli* ACC filaments will likely be needed to understand how acyl-ACP inhibits ACC.

When PlsB activity was artificially varied to mimic variations in phospholipid synthesis flux in our simple model featuring only ACC inhibition by acyl-ACP, simulated concentrations of both fatty acid and phospholipid synthesis intermediates closely followed our experimental data. This is strongly consistent with long-chain acyl-ACP inhibiting ACC during steady-state growth. We also found no indication that other proposed targets of acyl-ACP inhibition (specifically FabH and FabI) play any role in controlling the total amount of fatty acids produced, despite earlier *in vitro* evidence suggesting such interactions (22, 23).

Glycerol-3-phosphate synthesis

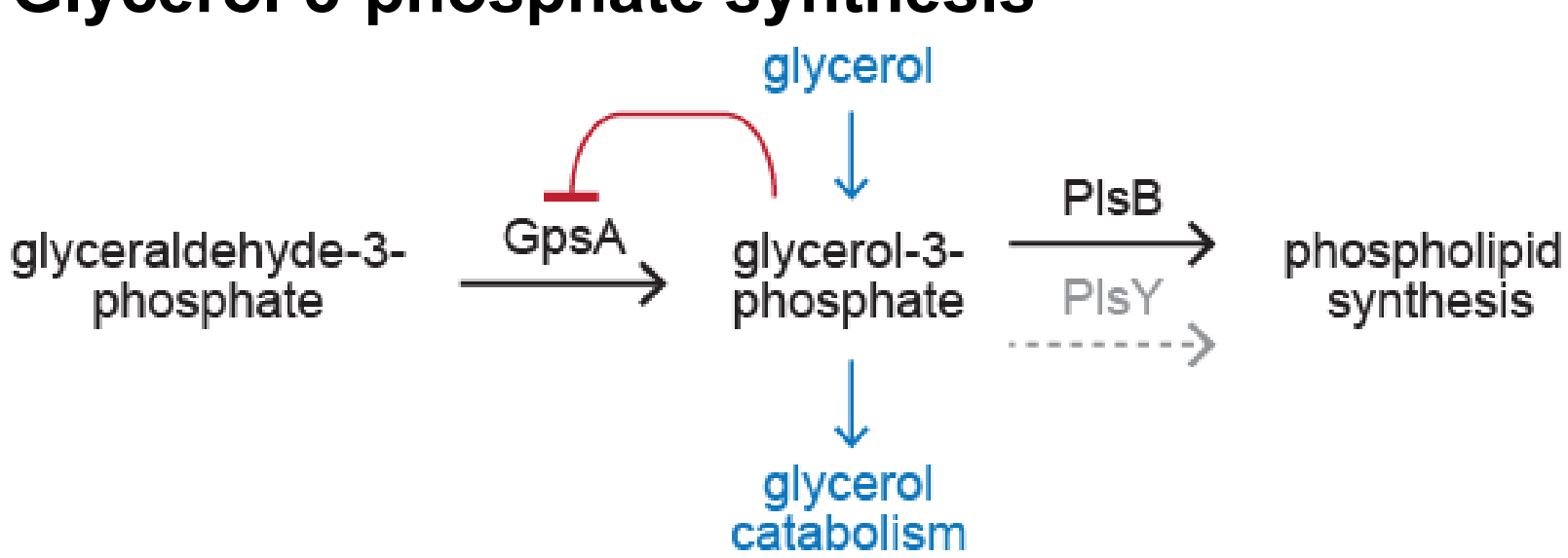

sn-glycerol-3-phosphate (G3P) provides the backbone of all phospholipids in *E. coli* as well as a headgroup for phosphatidylglycerol. G3P is synthesized from glycerone-3-phosphate by the glycerol-3-phosphate dehydrogenase enzyme GpsA. GpsA is product-inhibited by G3P (24). Cells expressing feedback-insensitive GpsA exhibit G3P concentration 12-fold higher than wild-

type, consistent with negative feedback (25). Later *in vitro* measurements obtained a K_I value of 4.4 μM (26). G3P concentrations are stable across most growth conditions but slightly increase with growth rate (27, 28). However, G3P is an intermediate of glycerol catabolism and is at least 20-fold more abundant in glycerol cultures (27). As glycerol cultures of *E. coli* do not exhibit any increase in phospholipid abundance, physiological concentrations of G3P do not influence membrane synthesis. Instead, GpsA maintains G3P at concentrations well above those required to sustain phospholipid synthesis by PlsB.

Phospholipid headgroup composition is maintained by negative feedback

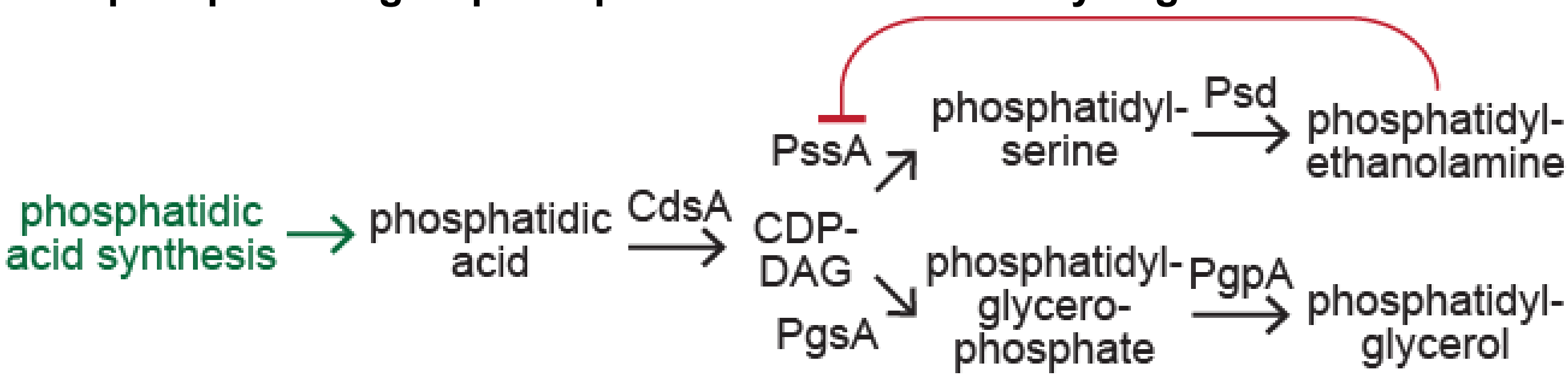

E. coli phospholipids come in three major species: phosphatidylethanolamine (PE) phosphatidylglycerol (PG), and cardiolipin (CL). PG comprises 15-20% of membrane phospholipids, while PE comprises 70-75% of membrane phospholipids. PG synthesis is initiated by CDP-diacylglycerol-glycerol-3-phosphate 3-phosphatidyltransferase PgsA, which displaces CMP with G3P (29) to produce phosphatidylglycerolphosphate (PGP). The terminal phosphate of PGP is removed by phosphatase PgpA to form PG (30), although the phosphatases PgpB and PgpC also contribute to PG synthesis (31, 32). PE synthesis is initiated by the transfer of serine to CDP-DAG by the phosphatidylserine synthase PssA to produce phosphatidylserine (PS) (33), which in turn is decarboxylated by phosphatidylserine decarboxylase Psd to produce PE (34).

The PE/PG ratio determines the electrostatic charge of the phospholipid membrane and influences lipid packing and membrane shape (35). The PE/PG ratio is determined by the relative activities of PssA and PgsA. Overexpression of either PssA or PgsA has very little effect on membrane composition (36, 37), suggesting the PE/PG ratio is maintained via a post-translational mechanism (38). The nature of the mechanism was first hinted at when PssA was found to associate with ribosomes (39), suggesting PssA might preferentially associate with anionic phospholipids such as PG. Saha et al. confirmed that PssA activity is impacted by anionic phospholipids (40). Further studies demonstrated that anionic phospholipids increase PssA association with the membrane (41, 42). Since headgroup modification reactions require

membrane association, increased association increases PE synthesis (40, 41). Dimerization of PssA may also contribute to regulation (43). Thus, membrane charge homeostasis is achieved via negative feedback: deficient PE activates PssA, while excess PE inhibits PssA.

UDP-*N*-acetyl glucosamine synthesis

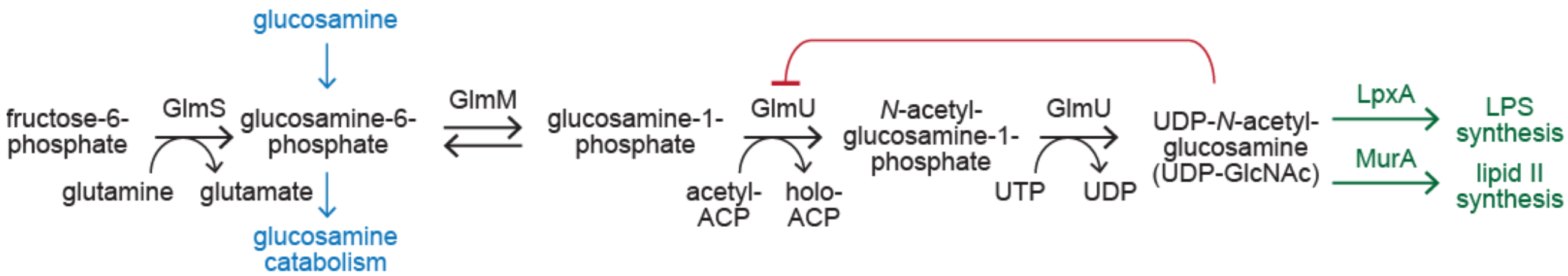

Both LPS and peptidoglycan synthesis rely upon UDP-*N*-acetyl glucosamine: it provides the anchoring headgroup for multiple lipids for lipid A and the core of peptidoglycan building block Lipid II. Glucosamine and *N*-acetyl glucosamine can be scavenged from the environment via a dedicated PTS system. In the absence of exogenous glucosamine, glucosamine-1-phosphate is produced from fructose-6-phosphate and glutamine by GlmS and GlmM. Next, the bifunctional enzyme GlmU transfers an acetyl group from acetyl-CoA (or acetyl-ACP, see below) and UDP to produce UDP-*N*-acetyl glucosamine (44–47). Remarkably, GlmU is inhibited by *N*-acetyl glucosamine but only weakly by UDP-*N*-acetyl glucosamine even at high concentrations (1 mM). As a consequence, UDP-*N*-acetyl glucosamine is very abundant in growing *E. coli* (2-9 mM) (48). This concentration likely drives the initial reaction of LPS biosynthesis, which is thermodynamically unfavorable (49).

Recently, an intriguing link between fatty acid synthesis and UDP-*N*-acetyl glucosamine synthesis was discovered. GlmU has long been thought to use acetyl-CoA as a substrate, although its K_M is rather high (600 μM) (50). Malonyl-ACP decarboxylase (MadA, also known as FabY and formerly as YiiD), has been recently discovered to generate acetyl-ACP from malonyl-ACP. This study found that the K_M of GlmU for acetyl-ACP is far lower (20 μM) (51), a strong indication that acetyl-ACP is the true physiological substrate for GlmU rather than acetyl-CoA. This may be important because acetyl-CoA concentrations are highly sensitive to the carbon source but are wholly uncorrelated with growth rate. Our measurements indicate that acetyl-ACP concentrations are far more stable than acetyl-CoA (27).

LPS synthesis

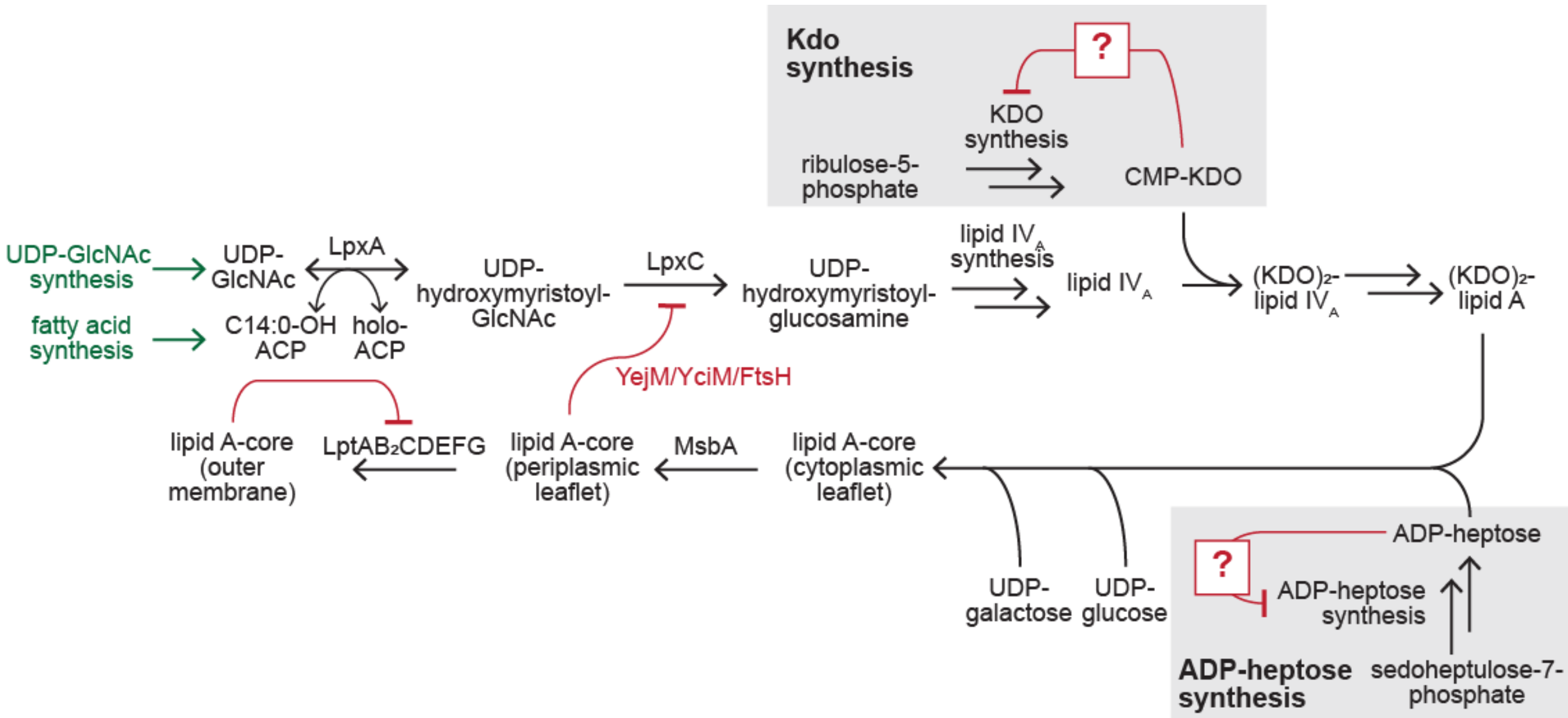

LPS are anchored by the glycolipid moiety Lipid A (a disaccharide functionalized with multiple fatty acids) bearing a core oligosaccharide. Phosphate groups attached to the disaccharide allow divalent ions to electrostatically bridge adjacent LPS. During synthesis within the cytoplasm and prior to transport to the outer membrane, LPS and its synthesis intermediates intercalate into the phospholipid inner membrane. LPS synthesis begins with the synthesis of Lipid A from intermediates of the fatty acid synthesis pathway. The first acylation of the *N*-acetyl glucosamine headgroup is catalyzed by acyl-ACP-UDP-*N*-acetylglucosamine O-acyltransferase LpxA, which transfers a C14:0-OH fatty acid from ACP to UDP-*N*-acetylglucosamine. Deacetylation of this intermediate by UDP-3-O-acyl-N-acetylglucosamine deacetylase (LpxC) commits the intermediate to the LPS biosynthesis pathway. Subsequent steps, which include several additional transfers of acyl groups, yield Lipid IV_A. Lipid IV_A is glycosylated using CMP-KDO and acylated to generate lipid A, which is further glycosylated with core saccharides to yield mature LPS. LPS is flipped from the cytoplasmic face of the inner membrane to the periplasmic face by MsbA. Periplasmic LPS modulates LpxC proteolysis via the YejM-YciM-FtsH system through a mechanism elucidated by multiple research groups (52–58) Finally, periplasmic LPS is extracted from the inner membrane and transported to the outer membrane by the Lpt system ($LptAB_2CDEFG$). As described in the main text, *in vitro* evidence suggests that LPS transport to the outer membrane is feedback-inhibited by outer membrane LPS.

In a pioneering application of metabolic models to envelope biogenesis, the effects of metabolic regulation on the LPS synthesis pathway has been extensively explored (59, 60). This

work highlighted the consequences of two envelope biogenesis pathways (LPS and phospholipid synthesis) originating from a common pathway (fatty acid synthesis). However, these models preceded the discovery of the homeostatic role of periplasmic LPS and its sensing by the YejM-YciM-FtsH system.

CMP-3-deoxy-D-*manno*-octulosonate (KDO) synthesis

KDO is attached to the glucosamine headgroup of lipid A and contributes the anchor point for the remaining moieties of the core oligosaccharide. KDO is synthesized via a 5-step pathway (61). KdsD isomerizes D-ribulose-5-phosphate to D-arabinose-5-phosphate. This is a reversible reaction (K_{eq} = 0.5) (62); thus the committed step of the pathway is likely catalyzed by KdsA, which condenses D-arabinose-5-phosphate with phosphoenolpyruvate to produce KDO-8-phosphate. KdsA is inhibited by its own product (K_I = 590 uM) but whether the final product of the pathway (CMP-KDO) inhibits KdsA is not known. KDO-8-phosphate is dephosphorylated by KdsC to produce KDO (63). Finally, KdsB activates KDO by transferring CMP from CTP. CMP-KDO is the substrate of WaaA, which transfers KDO to the LPS synthesis intermediate lipid IV_A to produce KDO-lipid A. To our knowledge, CMP-KDO concentrations have not been quantified in growing *E. coli*.

LPS core synthesis

The core sugars of LPS, glycero-β-D-*manno*-heptose, glucose, and galactose, are added as monomers in a specific order to $(KDO)_2$-lipid A by dedicated enzymes that use nucleotide diphosphate-activated monosaccharides UDP-glucose and UDP-galactose, which both share a 2-step synthesis starting from glucose-6-phosphate. UDP-glucose and UDP-galactose are also precursors for other cell components such as trehalose, and UDP-galactose is an intermediate in galactose catabolism. While the enzymes that produce both sugar nucleotides have been identified and characterized, no regulatory mechanisms have been shown. ADP-L-glycero-β-D-*manno*-heptose is exclusively used for LPS core saccharide synthesis and features a longer synthesis pathway that begins with isomerization of the central carbon metabolite seduheptulose 7-phosphate by the enzyme GmhA, followed by ATP-driven phosphorylation of the product by HldE. Subsequent steps are dephosphorylation by GmhB, addition of the ADP moiety by HldE, and a final isomerization step by RfaD. As far as we are aware, no regulatory mechanisms for this pathway have been discovered nor have concentrations of ADP-L-glycero-β-D-*manno*-heptose in growing *E. coli* been reported.

Peptidoglycan synthesis

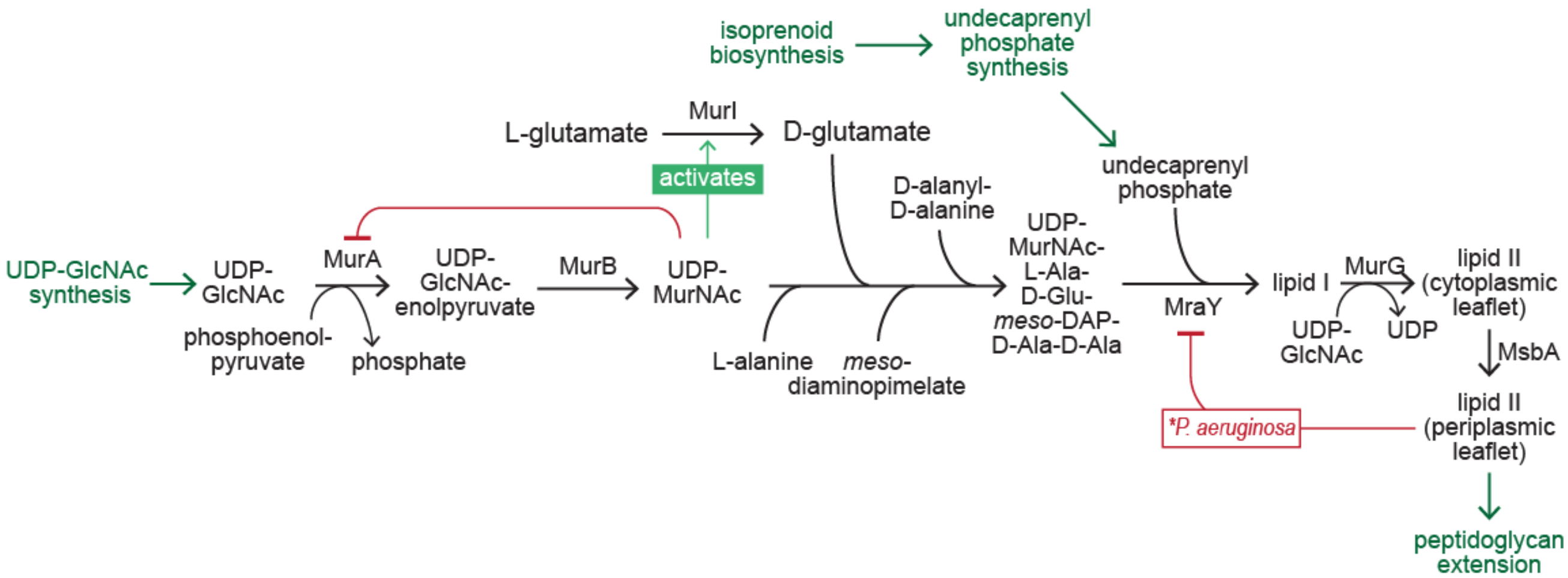

MurA is inhibited by UDP-MurNAc

After the synthesis of UDP-GlcNAc follows the two-step synthesis of UDP-N-acetyl-α-D-muramate (UDP-MurNAc). The first step, the transfer of enolpyruvyl from phosphoenolpyruvate to UDP-GlcNAc, is catalyzed by MurA (64). The MurA reaction is followed by a reduction step catalyzed by MurB, forming the intermediate UDP-MurNAc (65). This reaction is regulated to control the influx into peptidoglycan synthesis. UDP-MurNAc strongly inhibits MurA, suggesting that the irreversible step is controlled by subsequent steps that consume UDP-MurNAc. Inhibition of MurA by UDP-MurNAc is competitive with regard to phosphoenolpyruvate while inhibition towards UDP-GlcNAc appears to be mixed (66).

Glutamate racemase MurI is activated by UDP-MurNAc-L-Ala

The next step in the pathway is the addition of amino acids to the sugar moiety. The first step, addition of L-alanine to UDP-MurNAc to make UDP-N-acetylmuramoyl-L-alanine (UDP-MurNAc-L-Ala), is catalyzed by MurC (67). The next step is the addition of D-glutamate, catalyzed by MurD (68). . Isomerization of L-glutamate to the D form requires the racemase MurI (69). Interestingly, MurI is activated by the other substrate of MurD, UDP-MurNAc-L-Ala (70). In this way the racemization of glutamate is tightly linked to the available UDP-MurNAc-L-Ala. This regulation likely serves to prevent accumulation of D-glutamate in excess of pathway requirements rather than a mechanism for controlling the rate of lipid II synthesis.

Lipid II synthesis is feedback-inhibited across the inner membrane by periplasmic lipid II in *Pseudomonas aeruginosa*

Recently MraY from *Pseudomonas aeruginosa* was found to be allosterically inhibited by Lipid II (71). While *E. coli* and *P. aeruginosa* MraY have only a 67% identity match, the residue

implicated in lipid II binding is conserved and it therefore seems worthwhile to investigate if *E. coli* MraY is also inhibited by Lipid II. Such a mechanism would enable the enzymes of peptidoglycan synthesis that are located in the periplasm to control the cytoplasmic biosynthesis of lipid II and its precursors.

Supplemental References

1. Sorensen PG, Lutkenhaus J, Young K, Eveland SS, Anderson MS, Raetz CRH. 1996. Regulation of UDP-3-O-[R-3-hydroxymyristoyl]-N-acetylglucosamine Deacetylase in Escherichia coli: THE SECOND ENZYMATIC STEP OF LIPID A BIOSYNTHESIS . Journal of Biological Chemistry 271:25898–25905.

2. Hummels KR. 2025. The regulation of lipid A biosynthesis. Journal of Biological Chemistry 301:110556.

3. Ogura T, Inoue K, Tatsuta T, Suzaki T, Karata K, Young K, Su LH, Fierke CA, Jackman JE, Raetz CRH, Coleman J, Tomoyasu T, Matsuzawa H. 1999. Balanced biosynthesis of major membrane components through regulated degradation of the committed enzyme of lipid A biosynthesis by the AAA protease FtsH (HflB) in Escherichia coli. Mol Microbiol 31:833–844.

4. Clairfeuille T, Buchholz KR, Li Q, Verschueren E, Liu P, Sangaraju D, Park S, Noland CL, Storek KM, Nickerson NN, Martin L, Dela Vega T, Miu A, Reeder J, Ruiz-Gonzalez M, Swem D, Han G, DePonte DP, Hunter MS, Gati C, Shahidi-Latham S, Xu M, Skelton N, Sellers BD, Skippington E, Sandoval W, Hanan EJ, Payandeh J, Rutherford ST. 2020. Structure of the essential inner membrane lipopolysaccharide–PbgA complex. Nature 584:479–483.

5. Shu S, Tsutsui Y, Nathawat R, Mi W. 2024. Dual function of LapB (YciM) in regulating Escherichia coli lipopolysaccharide synthesis. Proc Natl Acad Sci U S A 121:e2321510121.

6. Lu W, Su X, Klein MS, Lewis IA, Fiehn O, Rabinowitz JD. 2017. Metabolite Measurement: Pitfalls to Avoid and Practices to Follow. Annu Rev Biochem 86:277–304.

7. Noga MJ, Büke F, van den Broek NJF, Imholz NCE, Scherer N, Yang F, Bokinsky G. 2020. Posttranslational Control of PlsB Is Sufficient To Coordinate Membrane Synthesis with Growth in Escherichia coli. mBio 11:e02703-19.

8. Li S-J, Cronan JE. 1992. The Genes Encoding the Two Carboxyltransferase Subunits of Escherichia coli Acetyl-CoA Carboxylase. J Biol Chem 267:16841–16847.

9. Guchhait RB, Polakis SE, Dimroth P, Stoll E, Moss J, Lane MD. 1974. Acetyl coenzyme A carboxylase system of Escherichia coli. Purification and properties of the biotin carboxylase, carboxyltransferase, and carboxyl carrier protein components. Journal of Biological Chemistry 249.

10. Williamson IP, Wakil SJ. 1966. Studies on the Mechanism of Fatty Acid Synthesis XVII. PREPARATION AND GENERAL PROPERTIES OF ACETYL COENZYME A AND MALONYL CO-ENZYME A-ACYL CARRIER PROTEIN TRANSACYLASES*THE JOURNAL OF Bro~oorcn~ CAEMIBTAY.

11. Oefner C, Schulz H, D'Arcy A, Dale GE. 2006. Mapping the active site of Escherichia coli malonyl-CoA-acyl carrier protein transacylase (FabD) by protein crystallography. Acta Crystallogr D Biol Crystallogr 62:613–618.

12. Jiang P, Cronan JE. 1994. Inhibition of Fatty Acid Synthesis in Escherichia coli in the Absence of Phospholipid Synthesis and Release of Inhibition by Thioesterase ActionJOURNAL OF BACTERIOLOGY.

13. Davis MS, Solbiati J, Cronan JE. 2000. Overproduction of acetyl-CoA carboxylase activity increases the rate of fatty acid biosynthesis in Escherichia coli. Journal of Biological Chemistry 275:28593–28598.

14. Cho H, Cronan JE. 1995. Defective export of a periplasmic enzyme disrupts regulation of fatty acid synthesis. Journal of Biological Chemistry 270:4216–4219.

15. Park WS, Shin KS, Jung HW, Lee Y, Sathesh-Prabu C, Lee SK. 2022. Combinatorial Metabolic Engineering Strategies for the Enhanced Production of Free Fatty Acids in Escherichia coli. J Agric Food Chem 70:13913–13921.

16. Lennen RM, Pfleger BF. 2012. Engineering Escherichia coli to synthesize free fatty acids. Trends Biotechnol https://doi.org/10.1016/j.tibtech.2012.09.006.

17. Davis MS, Cronan J. 2001. Inhibition of Escherichia coli acetyl coenzyme A carboxylase by acyl-acyl carrier protein. J Bacteriol 183:1499–1503.

18. Evans A, Ribble W, Schexnaydre E, Waldrop GL. 2017. Acetyl-CoA carboxylase from Escherichia coli exhibits a pronounced hysteresis when inhibited by palmitoyl-acyl carrier protein. Arch Biochem Biophys 636:100–109.

19. Park CK, Horton NC. 2019. Structures, functions, and mechanisms of filament forming enzymes: a renaissance of enzyme filamentation. Biophys Rev 11:927–994.

20. Meredith MJ, Lane MD. 1978. Acetyl-CoA carboxylase. Evidence for polymeric filament to protomer transition in the intact avian liver cell. Journal of Biological Chemistry 253:3381–3383.

21. Xu X, Silva de Sousa A, Boram TJ, Jiang W, Lohman JR. 2024. Active *E. coli* heteromeric acetyl-CoA carboxylase forms polymorphic helical tubular filaments https://doi.org/10.1101/2024.05.28.596234.

22. Heath RJ, Rock CO. 1996. Inhibition of-Ketoacyl-Acyl Carrier Protein Synthase III (FabH) by Acyl-Acyl Carrier Protein in Escherichia coli*J. Biol. Chem.

23. Heath RJ, Rock CO. 1996. Regulation of fatty acid elongation and initiation by acyl-acyl carrier protein in Escherichia coli. Journal of Biological Chemistry 271:1833–1836.

24. Cronan, JE, Rock CO. 2008. Biosynthesis of Membrane Lipids. EcoSal Plus 3.

25. Bells RM, Cronan AE. 1975. Mutants of Escherichia coli Defective in Membrane Phospholipid Synthesis PHENOTYPIC SUPPRESSION OF sn-GLYCEROL-3-PHOSPHATE ACYLTRANSFERASE K, MUTANTS BY LOSS OF FEEDBACK INHIBITION OF THE BIOSYNTHETIC sn-GLYCEROL-3-PHOSPHATE DEHYDROGENASE*THE JOURNAL OF Buxocrc~. CHEM~~RY.

26. Edgar JR, Bell RM. 1979. Biosynthesis in Escherichia coli of sn-Glycerol 3-Phosphate, a Precursor of Phospholipid. Palmitoyl-CoA inhibition of the biosynthetic sn-glycerol-3-phosphate dehydrogenase. J Biol Chem.

27. Noga MJ, Büke F, Van Den Broek NJF, Imholz NCE, Scherer N, Yang F, Bokinsky G. 2020. Posttranslational Control of PlsB Is Sufficient To Coordinate Membrane Synthesis with Growth in Escherichia coli. mBio https://doi.org/10.1128/mBio.

28. Radoš D, Donati S, Lempp M, Rapp J, Link H. 2022. Homeostasis of the biosynthetic E. coli metabolome. iScience 25:e104503.

29. Hirabayashi T, Larson TJ, Dowhan W. 1976. Membrane-Associated Phosphatidylglycerophosphate Synthetase from Escherichia coli: Purification by Substrate Affinity Chromatography on Cytidine 5'-Diphospho-1,2-diacyl-sn-glycerol Sepharose. Biochemistry.

30. Icho T, Raetz CRH. 1983. Multiple Genes for Membrane-Bound Phosphatases in Escherichia coli and Their Action on Phospholipid PrecursorsJOURNAL OF BACTERIOLOGY.

31. Funk CR, Zimniak L, Dowhan W. 1992. The pgpA and pgpB Genes of Escherichia coli Are Not Essential: Evidence for a Third Phosphatidylglycerophosphate PhosphataseJOURNAL OF BACTERIOLOGY.

32. Dillon DA, Wu W-I, Riedel B, Wissing JB, Dowhan W, Carman GM. 1996. The Escherichia coli pgpB Gene Encodes for a Diacylglycerol Pyrophosphate Phosphatase Activity*.

33. Raetz CRH. 1976. Phosphatidylserine synthetase mutants of Escherichia coli. Genetic mapping and membrane phospholipid composition. Journal of Biological Chemistry 251:3242–3249.

34. Hawrot E, Kennedy EP. 1975. Biogenesis of Membrane Lipids: Mutants of Escherichia coli with Temperature-Sensitive Phosphatidylserine Decarboxylase* (membrane function/phosphatidylethanolamine/psd mutants/phospholipid). Proc Nar Acad Sci USA 72:1112–1116.

35. Rowlett VW, Mallampalli VKPS, Karlstaedt A, Dowhan W, Taegtmeyer H, Margolin W, Vitrac H. 2017. Impact of Membrane Phospholipid Alterations in Escherichia coli on Cellular Function and Bacterial Stress Adaptation https://doi.org/10.1128/JB.

36. Tan Z, Khakbaz P, Chen Y, Lombardo J, Yoon JM, Shanks J V., Klauda JB, Jarboe LR. 2017. Engineering Escherichia coli membrane phospholipid head distribution improves tolerance and production of biorenewables. Metab Eng 44:1–12.

37. Ohta A, Waggoner K, Louie K, Dowhan W. 1981. Cloning of genes involved in membrane lipid synthesis. Effects of amplification of phosphatidylserine synthase in Escherichia coli. Journal of Biological Chemistry 256:2219–2225.

38. Zhang YM, Rock CO. 2008. Membrane lipid homeostasis in bacteria. Nat Rev Microbiol https://doi.org/10.1038/nrmicro1839.

39. Raetz CR, Kennedy EP. 1972. The association of phosphatidylserine synthetase with ribosomes in extracts of Escherichia coli. J Biol Chem 247:2008–2014.

40. Saha SK, Satomi N, Matsuzaki H, Shibuya I, Matsumoto K. 1996. A Regulatory Mechanism for the Balanced Synthesis of Membrane Phospholipid Species in Escherichia coli. ioscience, Biotechnology, and Biochemistry 116.

41. Linde K, Gröbner G, Rilfors L. 2004. Lipid dependence and activity control of phosphatidylserine synthase from Escherichia coli. FEBS Lett 575:77–80.

42. Salamon Z, Ran Lindblom G, Rilfors L, Linde K, Tollin G. 2000. Interaction of Phosphatidylserine Synthase from E. coli with Lipid Bilayers: Coupled Plasmon-Waveguide Resonance Spectroscopy Studies. Biophys J.

43. Lee E, Cho G, Kim J. 2024. Structural basis for membrane association and catalysis by phosphatidylserine synthase in Escherichia coliSci. Adv.

44. Mengin-Lecreulx D, Van Heijenoort J. 1996. Characterization of the essential gene glmM encoding phosphoglucosamine mutase in Escherichia coli. Journal of Biological Chemistry 271:32–39.

45. Chmarat H. 1985. Inhibition of Glucosamine Synthase by Bacilysin and AnticapsinJournal of General Microbiology.

46. Mengin Lecreulx D, vAN HEIJENOORT J, Walker JE, Gay NJ, Saraste M, Eberle AN. 1993. Identification of the glmU Gene Encoding N-Acetylglucosamine-1-Phosphate Uridyltransferase in Escherichia coli The physiological properties of the EcoURF-1 open reading frame, which precedes the gbnS gene at 84 min on the Escherichia coli chromosomeJOURNAL OF BACTERIOLOGY.

47. Wu HC, Wu TC. 1971. Isolation and Characterization of a Glucosamine-Requiring Mutant of Escherichia coli K-12 Defective in Glucosamine-6-Phosphate SynthetaseJOURNAL OF BACTERIOLOGY.

48. Bennett BD, Kimball EH, Gao M, Osterhout R, Van Dien SJ, Rabinowitz JD. 2009. Absolute metabolite concentrations and implied enzyme active site occupancy in Escherichia coli. Nat Chem Biol 5:593–599.

49. Anderson MS, Bull HG, Galloway$ SM, Kelly TM, Mohan S, Radikag K, Raetzn CRH. 1993. UDP-N-acetylglucosamine Acyltransferase of Escherichia coli - The first step of endotoxin biosynthesis is thermodynamically unfavourable. J Biol Chem.

50. Mengin Lecreulx D, JEAN vA HEIJENOORT A. 1994. Copurification of Glucosamine-1-Phosphate Acetyltransferase and N-Acetylglucosamine-1-Phosphate Uridyltransferase Activities of Escherichia coli: Characterization of the glmU Gene Product as a Bifunctional Enzyme Catalyzing Two Subsequent Steps in the Pathway for UDP-N-Acetylglucosamine SynthesisJOURNAL OF BACTERIOLOGY.

51. Whaley SG, Radka CD, Subramanian C, Frank MW, Rock CO. 2021. Malonyl-acyl carrier protein decarboxylase activity promotes fatty acid and cell envelope biosynthesis in Proteobacteria. Journal of Biological Chemistry 297:e101434.

52. Fivenson EM, Bernhardt TG. 2020. An essential membrane protein modulates the proteolysis of LpxC to control lipopolysaccharide synthesis in Escherichia coli. mBio 11:e00939-20.

53. Nguyen D, Kelly K, Qiu N, Misra R. 2020. YejM controls LpxC levels by regulating protease activity of the FtsH/YciM complex of Escherichia coli. J Bacteriol 202:1110–1128.

54. Guest RL, Guerra DS, Wissler M, Grimm J, Silhavy TJ. 2020. YejM modulates activity of the YciM/FtsH protease complex to prevent lethal accumulation of lipopolysaccharide. mBio 11:e00598-20.

55. Shu S, Mi W. 2022. Regulatory mechanisms of lipopolysaccharide synthesis in Escherichia coli. Nat Commun 13:4576.

56. Clairfeuille T, Buchholz KR, Li Q, Verschueren E, Liu P, Sangaraju D, Park S, Noland CL, Storek KM, Nickerson NN, Martin L, Dela Vega T, Miu A, Reeder J, Ruiz-Gonzalez M, Swem D, Han G, DePonte DP, Hunter MS, Gati C, Shahidi-Latham S, Xu M, Skelton N, Sellers BD, Skippington E, Sandoval W, Hanan EJ, Payandeh J, Rutherford ST. 2020. Structure of the essential inner membrane lipopolysaccharide–PbgA complex. Nature 584:479–483.

57. Klein G, Kobylak N, Lindner B, Stupak A, Raina S. 2014. Assembly of lipopolysaccharide in Escherichia coli requires the essential LapB heat shock protein. Journal of Biological Chemistry 289:14829–14853.

58. Mahalakshmi S, Sunayana MR, Saisree L, Reddy M. 2014. YciM is an essential gene required for regulation of lipopolysaccharide synthesis in Escherichia coli. Mol Microbiol 91:145–157.

59. Emiola A, George J, Andrews SS. 2015. A complete pathway model for lipid a biosynthesis in Escherichia coli. PLoS One 10.

60. Emiola A, Andrews SS, Heller C, George J. 2016. Crosstalk between the lipopolysaccharide and phospholipid pathways during outer membrane biogenesis in *Escherichia coli*. Proceedings of the National Academy of Sciences 113:3108–3113.

61. Smyth KM, Marchant A. 2013. Conservation of the 2-keto-3-deoxymanno-octulosonic acid (Kdo) biosynthesis pathway between plants and bacteria. Carbohydr Res. Elsevier Ltd https://doi.org/10.1016/j.carres.2013.07.006.

62. Meredith TC, Woodard RW. 2003. Escherichia coli YrbH is a D-arabinose 5-phosphate isomerase. Journal of Biological Chemistry 278:32771–32777.

63. Wu J, Woodard RW. 2003. Escherichia coli YrbI is 3-deoxy-D-manno-octulosonate 8-phosphate phosphatase. Journal of Biological Chemistry 278:18117–18123.

64. Marquardt JL, Siegele DA, Kolter R, Walsh CT, Trach K, Chapman JW, Piggot P, LeCoq D, Hoch JA, Bacteriol J. 1992. Cloning and Sequencing of Escherichia coli murZ and Purification of Its Product, a UDP-N-Acetylglucosamine Enolpyruvyl TransferaseJOURNAL OF BACTERIOLOGY.

65. Hid I, Anwar A, Veaovhg M. 1978. Purification of UDP-N-acetylenopyruvoylglucosamine reductase from Escherichia coli by affinity chromatography, its subunit structure and the absence of flavin as the prosthetic group. Can J Biochem.

66. Mizyed S, Oddone A, Byczynski B, Hughes DW, Berti PJ. 2005. UDP-N-acetylmuramic acid (UDP-MurNAc) is a potent inhibitor of MurA (enolpyruvyl-UDP-GlcNAc synthase). Biochemistry 44:4011–4017.

67. Liger D, Masson A, Blanot D, van Heijenoort J, Parquet C. 1995. Overproduction, Purification and Properties of the Uridine-diphosphate N-Acetylmuramate: l-alanine Ligase from Escherichia coli. Eur J Biochem 230:80–87.

68. PRATVIEL-SOSA F, MENGIN-LECREULX D, van HEIJENOORT J. 1991. Over-production, purification and properties of the uridine diphosphate N-acetylmuramoyl-l-alanine: d-glutamate ligase from Escherichia coli. Eur J Biochem 202:1169–1176.

69. Doublet P, Van Heijenoort J, Mengin-Lecreulx D, Brosius J, Dull TJ, Sleeter DD, Noller HFJB. 1992. Identification of the Escherichia coli murI Gene, Which Is Required for the Biosynthesis of D-Glutamic Acid, a Specific Component of Bacterial PeptidoglycanJOURNAL OF BACTERIOLOGY.

70. Doublet P, Van Heijenoort J, Mengin-Lecreulx D. 1994. The Glutamate Racemase Activity from Escherichia coli Is Regulated by Peptidoglycan Precursor UDP-TV-acetylmuramoyl-L-alanine1Biochemistry.

71. Marmont LS, Orta AK, Baileeves BWA, Sychantha D, Fernández-Galliano A, Li YE, Greene NG, Corey RA, Stansfeld PJ, Clemons WM, Bernhardt TG. 2024. Synthesis of lipid-linked precursors of the bacterial cell wall is governed by a feedback control mechanism in Pseudomonas aeruginosa. Nat Microbiol 9:763–775.
